# Multi-omics analysis of the tumor microenvironment after metastasis: advancing toward personalized immunotherapy and molecular targeted strategies

**DOI:** 10.3389/fimmu.2025.1648987

**Published:** 2025-09-23

**Authors:** Qing Gao, Huqiang Wu, Li Duan, Guanhua Lv, Dongmei Quan

**Affiliations:** ^1^ School of Life Sciences, Zhejiang Chinese Medicine University, Hangzhou, Zhejiang, China; ^2^ Department of Ophthalmology, The First Affiliated Hospital of Yunnan University of Chinese Medicine, Kunming, Yunnan, China; ^3^ Department of Rheumatology, The First Affiliated Hospital of Yunnan University of Chinese Medicine, Kunming, Yunnan, China; ^4^ Department of Gastroenterology, The Second Affiliated Hospital of Liaoning University of Traditional Chinese Medicine, Shenyang, China; ^5^ Second Clinical School, Liaoning University of Traditional Chinese Medicine, Shenyang, Liaoning, China

**Keywords:** metastatic tumor microenvironment, multi-omics, immune evasion, therapeutic resistance, precision medicine

## Abstract

The metastatic tumor microenvironment (TME) is a highly dynamic and heterogeneous ecosystem that plays a critical role in promoting cancer cell colonization, immune escape, and resistance to therapy. Recent advances in multi-omics technologies—including genomics, transcriptomics, epigenomics, proteomics, and metabolomics—have enabled a systems-level understanding of the molecular reprogramming that occurs in the TME following metastasis. In this review, we systematically summarize emerging findings from recent multi-omics studies that dissect cellular composition, signaling pathways, immune landscape, and metabolic rewiring within the metastatic TME. We highlight key molecular signatures and intercellular interactions that drive metastatic progression and therapy resistance. Furthermore, we discuss how integrative multi-omics data are being leveraged to identify actionable targets and to design novel immunotherapeutic and molecular precision strategies tailored to the metastatic niche. These insights provide a scientific rationale for the development of TME-targeted approaches in the treatment of advanced-stage cancers.

## 1 Introduction

Tumor metastasis remains the leading cause of cancer-related deaths, accounting for over 90% of cancer mortalities (1, 2). While early-stage cancers often have favorable prognoses through surgery and adjuvant therapies, the occurrence of metastasis significantly increases treatment complexity and drastically reduces patient survival rates (3). In addition to their invasiveness and migratory capabilities, tumor cells actively transform the local milieu at metastatic sites, creating a “metastatic niche,” as the cancer spreads (4–6). This metastatic microenvironment is composed of tumor cells, immune cells, fibroblasts, the vascular system, and various extracellular matrix components, collectively forming a complex ecosystem that supports tumor cell survival, proliferation, and immune evasion.

Traditional single-omics approaches, such as relying solely on genomic or transcriptomic analyses, often fall short in capturing the multidimensional interactions within the metastatic tumor microenvironment (7, 8). This is mainly because no single omics layer can provide a complete understanding of the many molecular regulatory mechanisms involved in tumor metastasis, such as genetic mutations, epigenetic alterations, protein expression control, and metabolic reprogramming (9, 10). A comprehensive picture of the metastatic tumor microenvironment is now possible thanks to the advent of multi-omics technologies that combine information from genomes, transcriptomics, epigenomics, proteomics, and metabolomics (11–13). In addition to illuminating the complex networks of communication between tumor cells, stromal cells, and immune cells in the surrounding area, this multi-dimensional molecular view also provides insight into the ever-changing functional states of immune cells and how they impact the therapeutic response.

Theoretically and technically, the development of immunotherapies and precision molecular-targeted medicines can be supported by the integrated application of multi-omics technologies (14–16). By identifying metastasis-specific molecular markers and key driver pathways, more targeted therapeutic strategies can be devised to overcome resistance and recurrence associated with conventional treatments (17–19). Further, by identifying patient-specific neoantigens and immune suppression mechanisms, multi-omics analysis can personalize immunotherapy, which in turn improves the efficacy of immunotherapies such immune checkpoint inhibitors (20–23). Therefore, a comprehensive elucidation of the multi-omics characteristics of the metastatic tumor microenvironment is essential for advancing therapeutic paradigms in late-stage cancers and for improving patient survival and quality of life.

The purpose of this article is to provide a comprehensive overview of the latest developments in multi-omics technologies that have been developed to better understand the intricate workings of the tumor microenvironment after metastasis, to discuss these technologies’ possible uses in molecularly targeted modeling and immunotherapy optimization, and to draw attention to the obstacles and opportunities that exist in the field of clinical translation. Our goal in directing this study is to help develop cancer precision treatment by shedding light on hitherto unexplored scientific questions and offering strategic backing for this field.

## 2 Multi-omics profiling of the tumor microenvironment after metastasis

### 2.1 Insights from genomics and transcriptomics

Genomic studies provide critical scientific evidence for revealing the molecular mechanisms underlying tumor metastasis. Through whole-genome sequencing (WGS) and targeted sequencing technologies, researchers have identified numerous key genes that frequently undergo mutations or copy number variations (CNVs) during the metastatic process (24, 25). These genes play crucial roles in tumor initiation, progression, and metastatic potential. For instance, TP53, a classical tumor suppressor gene, is highly mutated across various cancer types (26–28). Genomic instability is reduced when TP53 activity is lost because it causes cell cycle dysregulation and defective DNA damage repair pathways. The result is an increase in tumor cell migration, invasiveness, resistance to apoptosis, and proliferation (29, 30). Additionally, activating mutations in oncogenes such as KRAS and PIK3CA play pivotal roles in metastasis. KRAS mutations cause constitutive activation of the MAPK signaling pathway, promoting cell proliferation, survival, and motility (31, 32). Tumor cells have an advantage in their ability to invade and metastasize when PIK3CA mutations activate the PI3K/Akt signaling pathway, which controls cellular metabolism, proliferation, and cytoskeletal remodeling (33, 34). Abnormal activation of these pathways not only accelerates local tumor growth but also facilitates tumor cells breaching the basement membrane, entering the bloodstream or lymphatic system, and metastasizing to distant organs (35, 36). Another important group of genetic changes is copy number variation, which controls the amounts of tumor suppressor and oncogene gene expression by means of gene amplification and deletion (37, 38). Amplification of oncogenes can markedly enhance malignant phenotypes such as proliferation, apoptosis resistance, and angiogenesis, whereas deletion of critical tumor suppressors weakens cellular defense mechanisms, facilitating metastasis (39, 40). To add insult to injury, CNVs can affect how tumor cells interact with their surroundings by influencing how tumor cells adapt to and avoid immune cells, stromal cells, and the extracellular matrix. Genomic studies that systematically analyze gene mutations and copy number variations (CNVs) provide valuable information for developing tailored treatment strategies by shedding light on the molecular causes of tumor metastasis and identifying promising therapeutic targets and biomarkers.

Transcriptomic studies employing RNA sequencing (RNA-seq) have uncovered extensive and dynamic alterations in gene expression profiles within metastatic tumor tissues compared to their primary counterparts (41–43). Tumor cells adapt, survive, and prosper in distant microenvironments by sophisticated molecular reprogramming, which is reflected in these transcriptome changes. Metastatic tumor cells are able to avoid being destroyed by the immune system because they show a marked increase in the expression of pathways that regulate the immune system (44, 45). Key immune checkpoint molecules, such as PD-L1 (programmed death-ligand 1) and CTLA-4 (cytotoxic T-lymphocyte-associated protein 4), are frequently overexpressed, serving to suppress T cell activation and promote immune tolerance (46, 47). In addition, the tumor immune microenvironment is transformed by the recruitment of regulatory T cells (Tregs) and myeloid-derived suppressor cells (MDSCs), which allows for the increased expression of immunosuppressive cytokines like IL-10, TGF-β, and IL-6. This creates an immunosuppressive niche that promotes tumor survival and metastasis (48–51). At the same time, metastatic areas show a dramatic increase in genes related to angiogenesis. Neovascularization is driven by vascular endothelial growth factor (VEGF) family members such as VEGFA and VEGFC, which stimulate migration, proliferation, and new vessel formation in endothelial cells. This increased angiogenic activity sustains the growth and spread of the metastatic tumor mass by ensuring that it receives an appropriate amount of oxygen and nutrients (52–54). Moreover, the transcriptomic landscape of metastatic tumors shows pronounced upregulation of genes involved in extracellular matrix (ECM) remodeling, which is essential for tumor invasion and migration. Elevated expression of structural ECM components such as various collagen isoforms (e.g., COL1A1, COL3A1) accompanies increased levels of matrix metalloproteinases (MMPs), including MMP2, MMP9, and MMP14 (55, 56). By hydrolyzing ECM proteins, these proteolytic enzymes make it easier for physical barriers to break down and alter the tumor microenvironment in a way that cancer cells can invade more easily (57, 58). Additionally, the increased expression of integrins and other adhesion molecules supports enhanced tumor cell motility and interaction with stromal components. These transcriptome changes, when taken as a whole, show how tumor cells communicate with the stroma, immune cells, and vasculature around them in a way that promotes metastasis. Gaining a grasp of these alterations in gene expression can shed light on the processes of metastasis and identify possible treatment targets to halt the growth of metastasis.

**Figure 1:**
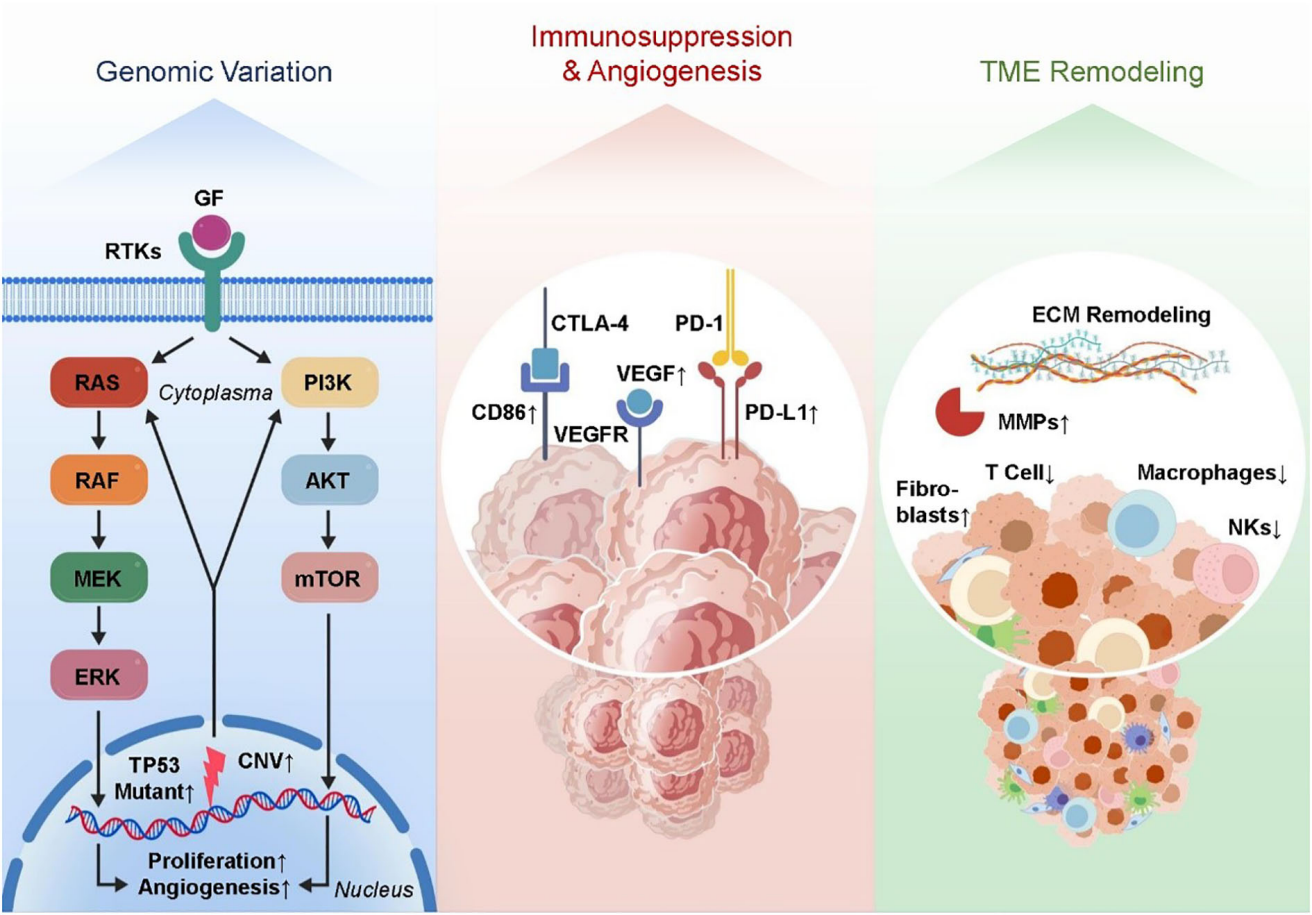
The regulatory genomic and transcriptomic landscape of the tumor microenvironment following metastasis. key mutations and CNVs activate oncogenic pathways. Transcriptomic changes include immune suppression, angiogenesis, and ECM remodeling. Single-cell RNA-seq reveals immune heterogeneity and enrichment of Tregs and M2 macrophages.

The advent of single-cell RNA sequencing (scRNA-seq) technology has profoundly deepened our understanding of the cellular heterogeneity and complexity within tumors and their associated microenvironments (59, 60). One advantage of single-cell RNA-seq over bulk RNA-seq is that it allows for high-resolution cell dissection, which is essential for identifying and characterizing different subpopulations of tumor cells, stromal components, and immune cells (61–63). This technology has become instrumental in unraveling the dynamic cellular ecosystem that drives tumor metastasis. The tumor microenvironment is functionally varied and extremely heterogeneous in metastatic situations, according to scRNA-seq. One example is the enrichment of regulatory T cells (Tregs) and M2-polarized macrophages within metastatic niches in breast cancer bone metastasis models. These cells have immunosuppressive features. A variety of immunosuppressive cytokines, including IL-10 and TGF-β, are released by these subsets of immune cells. These cytokines reduce the activity of cytotoxic T cells and make immunological escape easier (64–66). Moreover, M2 macrophages and Tregs contribute to angiogenesis by releasing pro-angiogenic factors like VEGF, thereby promoting neovascularization essential for metastatic tumor growth and sustenance (67, 68). Concurrently, during metastasis, tumor cells display extensive transcriptional plasticity. scRNA-seq studies have shown that tumor cells that have spread to other parts of the body increase the expression of genes related to drug resistance mechanisms, extracellular matrix remodeling, improved migratory potential, and epithelial-to-mesenchymal transition. A key component of both metastatic spread and treatment failure is the ability of cancer cells to invade distant regions and resist therapeutic stresses, which is achieved by transcriptional reprogramming (69, 70). Importantly, such single-cell resolution analyses allow the tracking of rare subpopulations, such as cancer stem-like cells or drug-tolerant persister cells, which may drive relapse and metastasis. Beyond descriptive profiling, transcriptomic data derived from scRNA-seq facilitate the discovery of prognostic biomarkers and predictive signatures for therapeutic response. Differential gene expression analyses can pinpoint gene modules tightly correlated with patient outcomes, enabling risk stratification and guiding clinical decision-making (42, 71, 72). Particularly, these data provide critical insights into the mechanisms of resistance to therapies such as immune checkpoint inhibitors (ICIs). Metastatic and immune evasion traits can be better understood by combining gene regulatory network analysis with the identification of critical transcription factors and signaling cascades. Metastatic tumors often activate NF-κB and STAT3 transcription factors, which lead to the activation of genes related to inflammation and maintain an immunosuppressive tumor microenvironment (73–75). Targeting these pathways holds promise to disrupt the metastatic niche and enhance therapeutic efficacy. Collectively, genomic and transcriptomic studies—especially at single-cell resolution—have markedly expanded our understanding of the molecular and cellular landscape of tumor metastasis (76, 77). Critical driver mutations, alterations in gene expression, and complicated cell-cell interactions within the metastatic ecology have been uncovered by these techniques. This information not only improves our understanding of tumor biology in general, but it also lays the groundwork for precision medicine approaches, such as improved immunotherapy regimens and new targeted medicines. **Figure 1** shows that one effective way to enhance cancer patients’ clinical results is to integrate multi-omics data at the single-cell level. This allows us to better understand tumor heterogeneity and the complexities of metastasis.

Although multi-omics technologies have demonstrated great potential in tumor metastasis research, significant advantages and limitations exist among different techniques, necessitating systematic comparison and analysis. Single-cell omics technologies, such as single-cell RNA sequencing, offer high resolution and finely reveal cellular heterogeneity, making them powerful tools for dissecting the complexity of tumors and their microenvironments (12, 78). It is challenging for single-cell omics to faithfully portray the geographical distribution of cells inside tissues and their physical interactions with nearby cells due to the frequent absence of spatial information. Spatial omics technologies, on the other hand, (e.g., spatial transcriptomics and spatial proteomics) maintain tissue structure, which allows for the mapping of spatial relationships between tumor cells and surrounding immune and stromal cells. This mapping provides crucial insights into microenvironment formation and intercellular signaling (16, 79). However, current spatial omics methods have certain limitations in spatial resolution, detection sensitivity, and data volume, and their high costs restrict widespread application. Furthermore, integrating different omics data faces multiple challenges, including heterogeneous data formats, batch effects, noise interference, and the complexity of biological interpretation. Effectively combining single-cell omics with spatial omics, balancing cellular functional states and spatial localization, remains a research hotspot and challenge. Meanwhile, the heterogeneity and dynamic nature of proteomics and metabolomics data add further complexity to data integration (80, 81). In summary, a deep understanding of the strengths and limitations of various omics technologies aids in the rational selection and optimization of research strategies. Moving forward, leveraging the synergistic advantages of multi-omics and developing efficient data integration and analysis methods will advance the study of the tumor metastatic microenvironment toward more precise and comprehensive insights, providing a robust scientific foundation for clinical translation.

### 2.2 Epigenomic and proteomic characteristics

**Figure 2:**
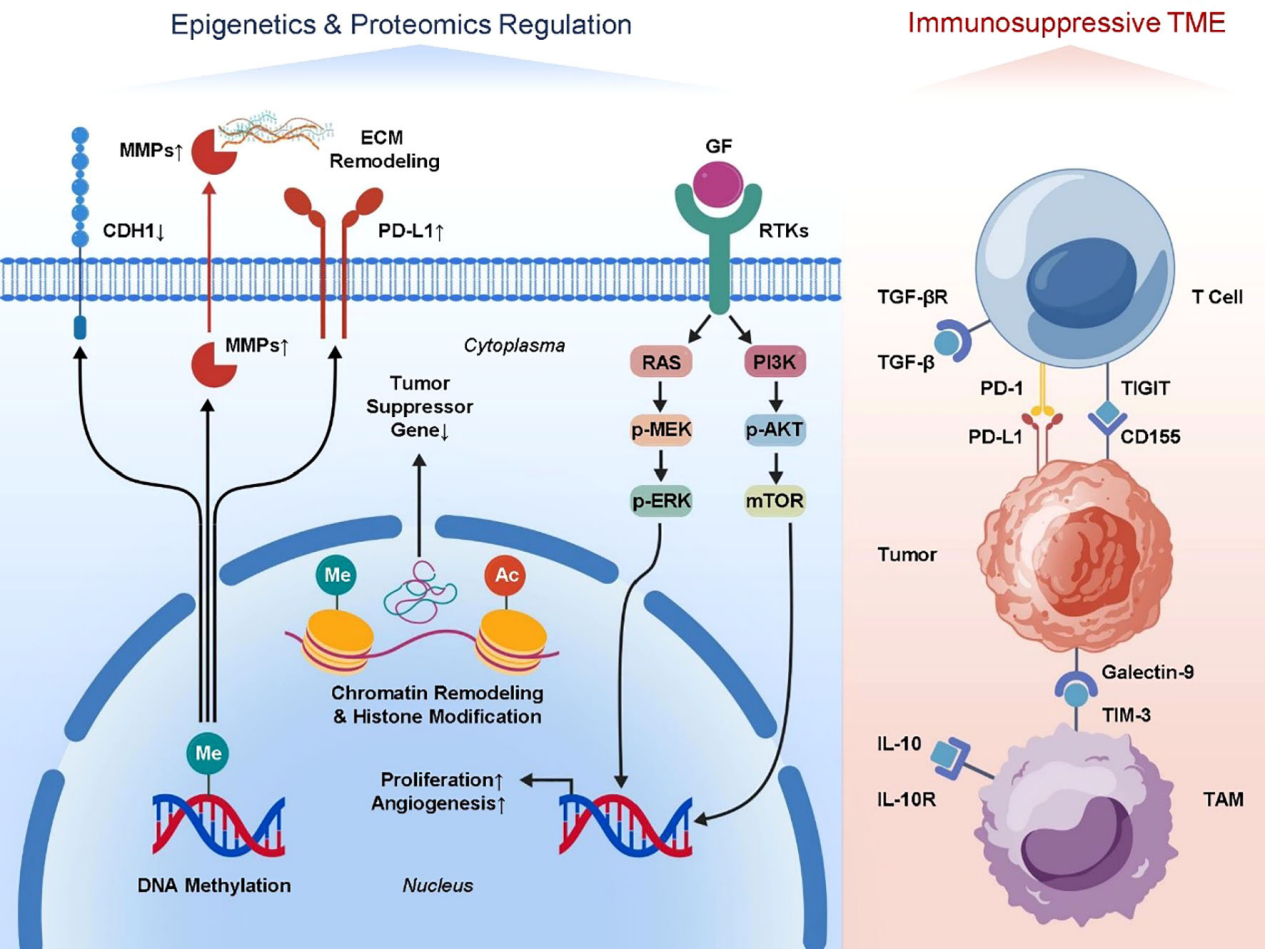
Mechanistic insights into epigenomic and proteomic regulation in metastatic tumors. DNA methylation and histone modifications alter gene expression. Key post-translational modifications regulate signaling pathways. Immune-related proteins and immunosuppressive cell proteomes show spatial heterogeneity, promoting metastasis and immune escape.

The molecular regulation of tumor metastasis is not limited to genetic mutations and changes in gene expression levels; epigenetic regulation also plays a crucial role. Epigenomics primarily involves mechanisms such as DNA methylation, histone modification, and chromatin remodeling (82–84). The spreading potential of tumor cells and the creation of the tumor microenvironment are impacted by these alterations, which dynamically regulate gene activity without changing the DNA sequence. Tumor metastasis is facilitated by DNA methylation in two ways (85–88). On one hand, hypomethylation of promoter regions of pro-metastatic genes can lead to their overexpression—for example, matrix metalloproteinases (MMPs) and genes related to epithelial–mesenchymal transition (EMT)—thus promoting tumor cell invasion and migration (89–91). On the other hand, hypermethylation-induced silencing of tumor suppressor genes, such as CDH1 (encoding E-cadherin), weakens intercellular adhesion and facilitates EMT, a critical step in the metastatic cascade (92, 93). Epigenetics also plays a role in regulating the expression of immunological checkpoint molecules. As an example, the tumor cell’s capacity to evade immune surveillance is impacted by the methylation state of the PD-L1 gene promoter, which controls its expression. Tumor cells can quickly adjust to changes in their microenvironment, control immunosuppressive pathways, and become more resistant to immunotherapy because epigenetic modifications are very malleable (94–97). Histone modifications—such as acetylation, methylation, and phosphorylation—also play significant roles in tumor metastasis. Histone acetylation is generally associated with gene activation, while histone methylation may either activate or repress gene expression, depending on the site and type of modification (98, 99). For example, trimethylation of histone H3 at lysine 27 (H3K27me3) is typically linked to gene silencing. Aberrant increases in H3K27me3 observed in some metastatic tumors suppress tumor suppressor gene expression and promote tumor progression (100–102). Chromatin remodeling complexes can alter chromatin structure and thus affect the accessibility of genes to transcription machinery, thereby mediating metastatic capability. Techniques such as ChIP-seq and whole-genome methylation sequencing have gradually uncovered the mechanistic roles of these modifications in shaping the metastatic microenvironment. Proteomics complements genomic and transcriptomic data, offering unique advantages in revealing protein expression and post-translational modifications (PTMs) (103, 104). In order to regulate the signaling and functional states of tumor cells, post-translational modifications (PTMs) such phosphorylation, ubiquitination, glycosylation, and methylation have a substantial impact on protein stability, activity, subcellular localization, and interactions. One example is the role of phosphorylation modifications in signaling pathway proteins in tumor cell migration, proliferation, and survival. These alterations are particularly important in the MAPK/ERK and PI3K/Akt pathways (105–107). Ubiquitination regulates protein degradation and signaling networks and is particularly important for tumor cells adapting to microenvironmental stress and immune evasion. Proteomic studies have also revealed complex signaling crosstalk between tumor cells and immune cells. For example, changes in the expression and modification states of immunosuppressive cytokines such as TGF-β and IL-10, and their receptors, modulate immune cell functions, promoting immune escape and tumor metastasis (108–110). Altered protein expression profiles in tumor-associated macrophages and regulatory T cells reflect the immunosuppressive status of the tumor microenvironment and provide new insights for discovering immunotherapy targets. In recent years, the emergence of spatial proteomics—especially techniques based on mass spectrometry—has enabled researchers to analyze protein expression and localization with high spatial resolution at the tissue section level. These technologies have revealed that the spatial distribution of specific protein modifications correlates with the degree of immune infiltration in metastatic lesions and the response to therapy, offering a more precise molecular basis for the implementation of personalized immunotherapy. In conclusion, the complex regulatory mechanisms of tumor metastasis and associated microenvironment are uncovered by combining epigenomics and proteome investigation. Both the control of gene expression and the modification of protein function are involved in these processes. To better understand tumor metastasis and its complex biological aspects, as well as to speed up the development of new immunotherapeutic techniques and molecularly targeted medications, integrative multi-omics studies are essential. These studies will provide patients with advanced cancer better treatment alternatives(**Figure 2**).

### 2.3 Metabolomic features

**Figure 3:**
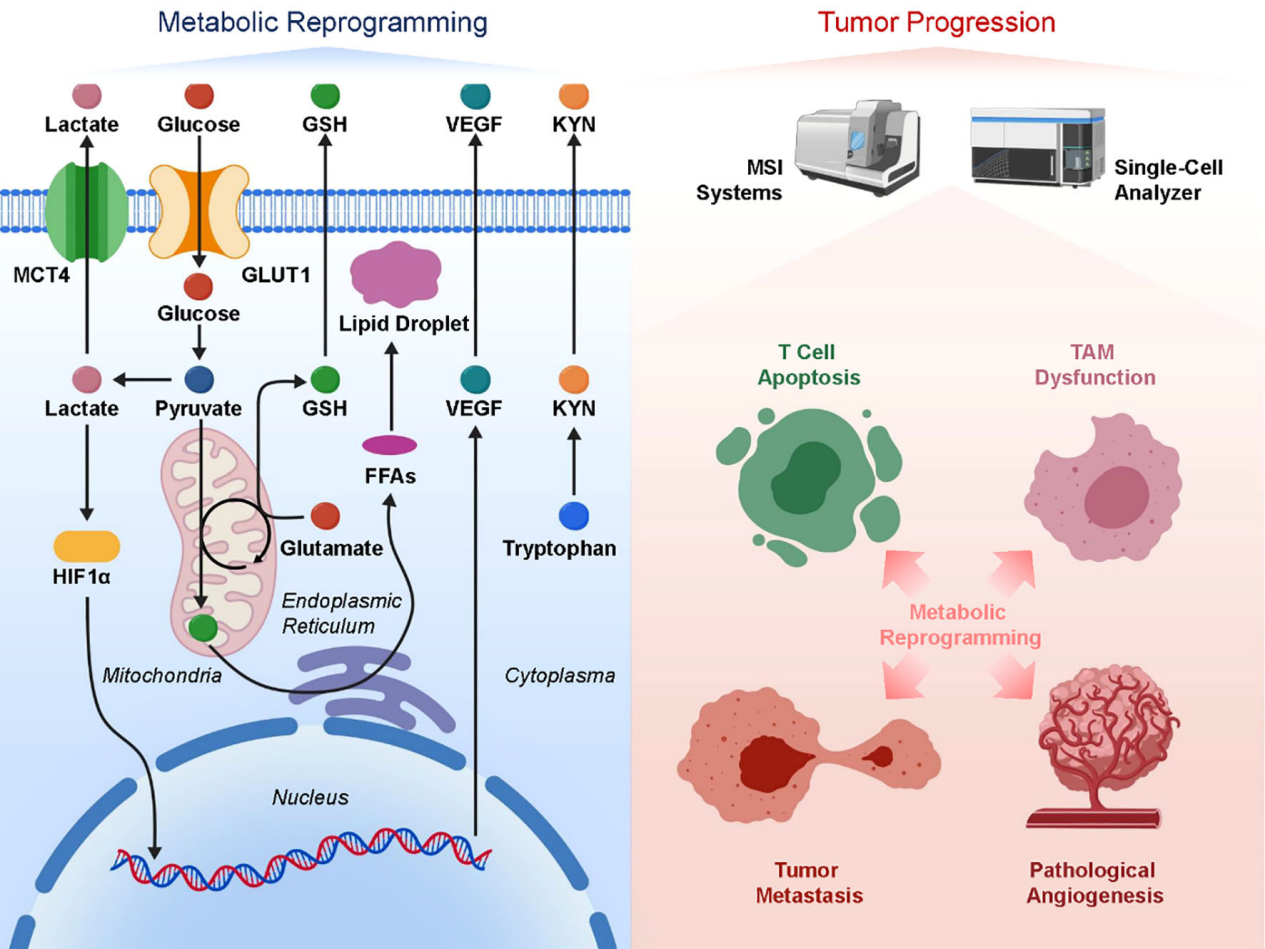
Metabolic adaptations in metastatic cancer and their effects on the tumor immune landscape. Enhanced glycolysis, lipid, and amino acid metabolism produce lactate and metabolites that suppress cytotoxic cells and promote immunosuppressive cells, aiding tumor migration, immune evasion, and angiogenesis.

Tumor metastasis involves not only alterations at the genomic and proteomic levels but also profound metabolic reprogramming. Within the metastatic microenvironment, tumor cells and surrounding supportive cells adjust metabolic pathways to meet the demands of rapid proliferation and adaptation to hostile conditions, while simultaneously shaping an immunosuppressive environment that facilitates sustained tumor growth and immune evasion (3, 111, 112). These metabolic regulators play a pivotal role in metastasis, as shown by metabolomics’ thorough profiling of metabolite alterations. Metastatic tumor cells exhibit elevated glycolysis, a metabolic characteristic known as the Warburg effect, which is highly noticeable (113, 114). Even under aerobic conditions, tumor cells preferentially utilize anaerobic glycolysis to generate energy, producing large amounts of lactate. The accumulation of lactate acidifies the tumor microenvironment, suppressing the activity of effector immune cells such as cytotoxic T lymphocytes and natural killer cells, while promoting the recruitment and polarization of immunosuppressive cells like regulatory T cells and tumor-associated macrophages (TAMs), thereby establishing an immune “cold” environment (115, 116). Moreover, lactate also induces angiogenesis, supporting the blood supply of metastatic lesions and enhancing tumor cell dissemination and survival (117, 118). Another important factor in tumor spreading is the reprogramming of lipid metabolism. Metastatic tumor cells speed up their migration and proliferation by acquiring the energy and membrane components they need through fatty acid production and oxidation. Fatty acid and cholesterol abnormal buildup regulates signaling pathway activation, improves cell motility, and promotes epithelial-mesenchymal transition (119, 120). Additionally, lipid metabolism regulates immune cell function—for example, TAMs promote immunosuppressive states via lipid-mediated signaling, contributing to immune escape. Changes to the metabolism of amino acids are just as important as those involving glucose and lipids. Tumor cells are able to resist oxidative stress because of improved glutamine uptake and metabolism, which supply nitrogen supplies necessary for biosynthesis and aid in regulating antioxidant capability. Tumor immune microenvironment modulation is thought to be mostly mediated by tryptophan metabolism, which promotes immunological tolerance and immune evasion through activation of the indoleamine 2,3-dioxygenase (IDO) pathway. Metabolomic research has also shown that immunotherapy effectiveness is strongly correlated with metabolic alterations in the metastatic microenvironment. Tumor cells deplete essential nutrients such as glucose and amino acids through metabolic competition, impairing the function of tumor-infiltrating lymphocytes and limiting the effectiveness of immune checkpoint inhibitors (120–122). As a result, targeting metabolism has emerged as a novel approach for combination immunotherapy. For example, inhibitors of lactate dehydrogenase (LDH) and regulators of fatty acid metabolism, when combined with immune checkpoint blockade, have shown enhanced anti-tumor efficacy in preclinical models. Researchers have recently been able to study metabolic heterogeneity at spatial and cellular resolutions using cutting-edge methods like mass spectrometry imaging (MSI) and single-cell metabolic analysis. This has led to a better understanding of how metabolic cooperation among cell types within metastatic lesions promotes tumor progression. Precision in detecting metabolic problems and creation of individualized metabolic intervention plans are both greatly facilitated by these technological advancements. In summary, metabolic reprogramming in metastatic tumors not only fulfills the energy demands of growth and dissemination but also regulates the immune microenvironment through multiple mechanisms, facilitating immune escape. Integrating metabolomic data with multi-omics approaches will enhance our understanding of tumor biology and support the development of metabolism-targeted combination therapies, ultimately improving the prognosis of patients with advanced cancer (**Figure 3**).

To provide a clear overview of multi-omics approaches in the metastatic TME, **Table 1** summarized major methods, representative assays, key findings, and clinical applications. Genomics identifies metastasis-associated mutations and clonal evolution, guiding targeted therapy; transcriptomics reveals immune remodeling and stromal heterogeneity, informing immunotherapy; proteomics and metabolomics capture metastasis-specific signaling and metabolic adaptations, offering biomarkers and therapeutic targets; epigenomics uncovers regulatory mechanisms in immune and stromal cells; and multi-omics integration highlights interactions and heterogeneity across tumor, immune, and stromal compartments. This table illustrates the translational value of multi-omics in precision medicine for metastatic cancer.

**Table 1:** Multi-omics approaches in the metastatic tumor microenvironment (TME).

Omics approach	Representative assays	Key findings in metastatic TME	Clinical utility
Genomics	Whole-exome sequencing (WES), targeted gene panels, ctDNA sequencing	Identification of metastasis-associated drivers (e.g., TP53, KRAS), clonal evolution, mutational signatures	Biomarker discovery for prognosis; stratification for targeted therapy
Transcriptomics	Bulk RNA-seq, single-cell RNA-seq, spatial transcriptomics	Immune remodeling (exhausted T cells, immunosuppressive macrophages), stromal heterogeneity, site-specific gene expression programs	Patient stratification for immunotherapy; prediction of immune checkpoint response
Proteomics	Mass spectrometry–based proteomics, CyTOF	Altered signaling pathways, cytokine/chemokine networks, immune checkpoint protein expression in metastases	Identification of therapeutic targets; biomarker panels for treatment monitoring
Epigenomics	ATAC-seq, bisulfite sequencing, ChIP-seq	Epigenetic reprogramming of immune and stromal cells, enhancer remodeling promoting metastasis	Development of epigenetic therapies; patient selection for combination strategies
Metabolomics	LC-MS/MS, NMR spectroscopy	Metabolic adaptation of metastatic niches (e.g., hypoxia-driven rewiring, lactate accumulation)	Predicting drug resistance; targeting metabolic vulnerabilities
Multi-omics integration	Computational modeling, network analysis	Cross-talk between immune, stromal, and tumor compartments; spatial and temporal heterogeneity	Precision medicine approaches; identification of combination therapy strategies

## 3 Implications for molecular targeting and immunotherapy

### 3.1 Optimizing molecular targeting models

Integrating data from genomes, transcriptomics, epigenomics, proteomics, and metabolomics to build accurate and dynamic molecular targeting models is a state-of-the-art approach in cancer treatment research, thanks to the fast development of multi-omics technology. Offering a more evidence-based and individualized foundation for molecular targeted therapy, this integrative approach uncovers the genetic and phenotypic traits of tumor cells while simultaneously delving deeper into the intricate networks of interactions between tumors and their microenvironment (11, 13). First, the integration of multi-omics data allows for the systematic capture of tumor heterogeneity. Genetic mutations, gene expression changes, epigenetic modifications, and protein functional states often vary significantly between patients and even among different regions of the same tumor. By integrating data across these dimensions, it becomes possible to accurately identify key molecular markers and regulatory networks driving tumor progression and metastasis. For example, targeted kinase inhibitors can be developed by merging genomic mutation data with transcriptome profiles, which can reveal overexpression or activating mutations in specific kinase genes. Theoretically, epigenetic data can aid in the creation of inhibitors of epigenetic enzymes by revealing aberrations in DNA methylation or histone changes that mute critical tumor suppressor genes (123, 124). Second, network analysis driven by multi-omics data can simulate signal transduction pathways between tumor cells, immune cells, and stromal cells, capturing dynamic intercellular communication. For instance, integrating proteomics data with transcriptomic profiles of immune cell infiltration can help identify key signaling pathways involved in immune evasion, such as the upregulation mechanisms of immune checkpoints like PD-1/PD-L1 and CTLA-4, thereby informing strategies for immunomodulatory molecular targeting. In addition, Metabolomics data can shed light on the ever-changing nutritional and metabolic product levels within tumor microenvironments, allowing for the identification of metabolic enzymes as possible therapeutic targets and providing necessary information for the development of integrated molecular targeting and metabolic intervention approaches. Integrating data from several omics studies has also become much smarter and more efficient with the help of machine learning and artificial intelligence. The development of individualized treatment programs can be facilitated by building prediction models using multi-omics features, which allow for the accurate forecasting of patient reactions to different targeted medications. For example, using data on a patient’s tumor mutation profile, protein expression levels, and metabolic status, the model can identify the most likely effective drug combinations, avoiding ineffective treatments and drug side effects. Moreover, multi-omics integration facilitates drug repurposing and the discovery of novel targets. Through horizontal comparisons and longitudinal tracking of large-scale patient data, previously overlooked molecular factors can be identified as critical players in specific metastatic types, driving the development of novel targeted drugs. Combined with clinical data, such models can also evaluate mechanisms of resistance to targeted therapy, guiding the design of second-line or combination treatment strategies to overcome therapeutic resistance. In summary, the core of optimizing molecular targeting models lies in comprehensively integrating multi-dimensional molecular information to reveal dynamic changes in the tumor microenvironment and multilayer regulatory mechanisms. This approach not only enhances the precision and efficacy of targeted therapies but also paves new pathways for personalized cancer treatment. As the volume of multi-omics data and computational capabilities continue to grow, systems biology-based molecular targeting models are poised to drive revolutionary advances in advanced cancer therapy.

### 3.2 Enhancing the efficacy of immunotherapy

Immunotherapy, particularly immune checkpoint inhibitors (ICIs), has emerged as a breakthrough in the treatment of various cancers. However, the clinical efficacy of ICIs shows significant heterogeneity across patients, with some individuals exhibiting no response or developing resistance (125, 126). Immunotherapy has progressed from empirical methods toward precision and tailored tactics, thanks to the integrated application of multi-omics technologies, which provide powerful tools for thoroughly studying the tumor immune milieu and its dynamic evolution (127–129). First, multi-omics data facilitate the identification and validation of immune-related biomarkers. Genomic sequencing can reveal the generation of tumor neoantigens—mutant tumor-specific antigens critical for eliciting T cell-mediated immune responses. By integrating transcriptomic and proteomic data, researchers can identify immunogenic proteins highly expressed on the tumor cell surface, guiding personalized cancer vaccine development and neoantigen-targeted therapies (130–132). Moreover, by analyzing epigenomes, we can learn how immune checkpoint genes (such as PD-L1 and CTLA-4) are regulated, which aids in evaluating immunosuppressive pathways and gives molecular proof for the use of checkpoint inhibitors (133, 134). Second, insights into T cell functionality and exhaustion, derived from multi-omics data, are pivotal for assessing immunotherapy responsiveness (135–137). Transcriptomic analyses enable detailed profiling of tumor-infiltrating lymphocyte (TIL) subpopulations and their activation states, identifying exhausted T cell subsets that express markers such as PD-1, LAG-3, and TIM-3 (138, 139). Potential targets for immune cell reactivation have been identified by proteomics, which provides additional validation of the expression levels and functional states of these surface molecules. Metabolomics research also shows that metabolites (such as lactate and adenosine) produced by tumors inhibit T cell activity, which can guide tactics that integrate metabolic regulation with immunological activation. Thirdly, immunotherapy resistance mechanisms can be better understood with the use of multi-omics methods. Immune evasion, antigen presentation abnormalities, and pathway activation are some of the failure-related variables that can be identified by tracking multi-omics alterations in tumors and immune cells before and after therapy. For example, genomic and transcriptomic data may reveal mutations in β2-microglobulin (B2M) that lead to antigen presentation loss, while epigenetic alterations can result in the downregulation of immune checkpoint targets. Uncovering these resistance mechanisms supports the rational design of combination therapies involving immune stimulators, epigenetic modulators, or metabolic interventions to overcome the limitations of monotherapy (140, 141). Furthermore, multi-omics technologies contribute to the development of immune-based stratification models for patient selection and efficacy prediction. By integrating tumor mutational burden (TMB), immune cell infiltration levels, immune gene expression profiles, and metabolic states, such models can predict a patient’s likelihood of responding to ICIs, thereby reducing unnecessary side effects and financial burden. The combination of multi-dimensional biomarkers also provides tools for real-time monitoring of treatment response and early detection of relapse. Lastly, immunometabolic crosstalk has become a research hotspot. Tumor metabolic reprogramming not only supports tumor cell survival but also modulates immune cell function through its metabolites. For instance, enhanced glycolysis in tumor cells leads to lactate accumulation, which suppresses the activity of effector T cells and natural killer cells. Multi-omics analyses help unravel these complex metabolic-immune networks, offering a scientific foundation for the design of immunometabolic combination therapies—such as using metabolic enzyme inhibitors in conjunction with immune checkpoint inhibitors to enhance antitumor immune responses. In summary, the integrated application of multi-omics technologies has deepened our understanding of the tumor immune microenvironment and provides valuable insights for optimizing immunotherapy strategies. With continued advancements in data analysis methods and bioinformatics tools, multi-omics-driven personalized immunotherapy is expected to significantly improve treatment response rates and survival outcomes for patients with advanced cancers.

## 4 Progress of existing clinical research

In recent years, clinical research integrating multi-omics data to understand and treat metastatic tumors has made significant strides. Numerous clinical trials have incorporated genomic, transcriptomic, proteomic, and metabolomic analyses to stratify patients, predict therapeutic responses, and identify novel biomarkers for personalized treatment. For example, tumor mutation burden (TMB) and specific gene expression profiles have been employed as predictive biomarkers to select patients likely to benefit from immune checkpoint inhibitors (ICIs), improving the efficacy of immunotherapy in metastatic cancers. Clinical trials such as KEYNOTE-158 and CheckMate-227 have demonstrated the utility of these biomarkers in guiding patient selection (142, 143). Additionally, targeted therapies guided by genomic alterations, such as EGFR mutations in non-small cell lung cancer and HER2 amplifications in breast cancer, have shown improved outcomes in metastatic settings (144–147). Ongoing studies are expanding the application of proteomic and metabolomic profiling to uncover resistance mechanisms and to design combination therapies. Moreover, integrated multi-omics approaches have been applied in clinical trials to monitor treatment response dynamically and to understand immune evasion mechanisms. For instance, the use of circulating tumor DNA (ctDNA) combined with proteomic markers enables real-time assessment of tumor evolution and therapeutic resistance (148, 149). Despite these advances, challenges remain in translating multi-omics findings into routine clinical practice, including data standardization, cost, and clinical validation. Nevertheless, the ongoing clinical research efforts are progressively bridging these gaps, paving the way for more precise and effective personalized therapies for patients with metastatic cancers.

## 5 Translational potential and clinical applications

The application of multi-omics technologies in clinical oncology is gradually maturing, significantly advancing the development of precision medicine. Large public databases such as The Cancer Genome Atlas (TCGA) and the Clinical Proteomic Tumor Analysis Consortium (CPTAC) integrate genomic, transcriptomic, proteomic, and clinical data from thousands of tumor samples, providing invaluable resources for researchers and clinicians. These databases not only reveal the molecular heterogeneity of various cancers but also aid in identifying potential therapeutic targets and biomarkers. With continuous advancements in omics technologies, an increasing number of clinical trials have begun incorporating multi-omics data into patient stratification and therapeutic response prediction. For example, some clinical trials select patients eligible for immune checkpoint inhibitor therapy based on tumor mutation burden (TMB), immune gene expression profiles, or specific metabolic markers. Such omics-based precision stratification not only improves treatment efficacy but also reduces adverse effects and the economic burden associated with ineffective therapies. However, several challenges remain in the clinical application of multi-omics. First, data standardization is an urgent issue. Differences in sequencing platforms, sample processing workflows, and data analysis methods across laboratories compromise data consistency and reproducibility. In order to achieve interoperability across platforms and centers, it is essential to establish uniform bioinformatics pipelines and common quality control techniques. Second, there are substantial challenges to clinical interpretation due to the complexity and high dimensionality of omics data. Single indicators fall short in properly capturing the complex networks formed by interactions across many omics layers in tumor tissues, which contain heterogeneous cell types. Deep data mining and pattern recognition made possible by AI and ML are essential components of the multidisciplinary effort needed to convert these complicated datasets into useful biomarkers or tools for clinical decision-making. Also, omics signatures must be validated in clinical settings. Lacking large-scale, multi-center clinical validation, many omics results are still in the preliminary discovery phase. The clinical application of omics biomarkers requires rigorous validation to ensure sensitivity, specificity, and predictive accuracy. Furthermore, transforming complex multi-omics signatures into simple, rapid, and cost-effective clinical assays is an important direction for broader implementation. Finally, ethical and privacy concerns must not be overlooked. Strict adherence to rules and regulations regarding data storage, distribution, and use is essential when dealing with omics data because of the large amounts of personally identifiable genetic information that is typically involved. In order to advance precision oncology, it is crucial to protect patient privacy while encouraging appropriate data consumption. Multi-omics analyses have identified numerous potential biomarkers and therapeutic targets in the metastatic TME, providing valuable insights into tumor progression and the development of precision therapies (150). However, the functional validation of the majority of these candidate molecules remains insufficient, limiting their clinical translational potential. *In vitro* cell lines and three-dimensional organoid models, *in vivo* animal models such as patient-derived xenografts (PDX), and clinical cohort association studies are key approaches for validating multi-omics discoveries. Some studies have employed *in vitro* functional assays to verify the roles of candidate molecules in cell proliferation, migration, and immune regulation (151, 152). Moreover, PDX models are widely used to recapitulate the biological characteristics of metastatic tumors, providing important evidence for the *in vivo* functions of molecular targets (153, 154). Clinical cohort analyses, based on large-scale sample databases, have validated the prognostic value and treatment response associations of these candidate molecules. Overall, there is great potential for multi-omics technologies in clinical oncology. However, there are several problems that need to be addressed during translation, including issues with standardization, data interpretation, clinical validation, and ethical security. Precision diagnosis and therapy made possible by multi-omics will bring in a new age in cancer treatment, thanks to ongoing interdisciplinary collaboration, technical progress, and the creation of regulatory frameworks.

## 6 Conclusion and future perspectives

The rapid development of multi-omics technologies is profoundly reshaping our understanding of the tumor metastatic microenvironment and influencing therapeutic strategies. The intricate molecular pathways and immunological regulatory networks inside metastatic tumor microenvironments can be better understood by combining information from the genome, epigenome, transcriptome, proteome, and metabolome. Not only does this improve our knowledge of tumor biology, but it also lays the groundwork for developing more targeted, efficient, and individualized methods of treatment. In the field of immunotherapy in particular, the integration of multi-omics data facilitates decoding of immune evasion mechanisms, immune cell dynamics, and interactions with tumor cells, greatly promoting the discovery of novel immunotherapeutic targets and combination strategies. Future research should focus on several key areas: First, developing more efficient and intelligent tools for multi-omics data integration and analysis is essential. Given the vast volume and structural complexity of omics data, leveraging cutting-edge technologies such as machine learning and artificial intelligence to achieve efficient data integration, deep mining, and dynamic model construction is foundational to advancing the field. Establishing unified platforms capable of handling multi-dimensional data will enable the full exploitation of complementary information across omics layers and facilitate the discovery of clinically meaningful biomarkers and therapeutic targets. Second, systematic clinical validation of multi-omics biomarkers is a core component of translational medicine. Future efforts must involve more large-scale, multi-center clinical trials to rigorously evaluate biomarker stability, sensitivity, and predictive power. Moreover, emphasis should be placed on translating omics discoveries into clinically accessible, cost-effective diagnostic tools to enable widespread application in real-world healthcare settings and fulfill the promise of precision medicine. Third, promoting interdisciplinary and inter-institutional collaboration is essential to accelerating the clinical translation of multi-omics technologies. The heterogeneity and complexity of tumors mean that no single institution can address all key issues independently. Multi-center cooperation not only provides access to diverse sample resources but also facilitates the unification of research standards and methodologies, enhancing the generalizability and credibility of findings. In addition, establishing open-access multi-omics databases and bioinformatics platforms to foster data sharing and collaboration will significantly drive innovation and development in precision oncology. Finally, ethical and privacy concerns remain critical. To strike a compromise between protecting patients’ privacy and making responsible use of data, strong data protection systems are required to accommodate the massive amounts of multi-omics data being created and used. For omics technologies to be used consistently and in a way that is acceptable to society, lawmakers, clinical researchers, and tech developers must collaborate to establish rules and recommendations based on solid science. In conclusion, multi-omics technologies offer a new perspective and pathway for investigating metastatic tumor microenvironments and advancing precision therapies. As analytical tools improve, clinical validation progresses, and collaborative efforts expand, multi-omics will play an increasingly central role in cancer immunotherapy and targeted therapy. This advancement promises to usher in a new era of more precise, effective, and personalized cancer treatment, offering new hope and improved quality of life for patients with advanced-stage cancer.
